# Cross-cultural adaption of the Knee injury and Osteoarthritis Outcome Score (KOOS) into Punjabi for knee injury and osteoarthritis patients in Canada

**DOI:** 10.1186/s12891-025-08870-y

**Published:** 2025-07-04

**Authors:** Nitya Suryaprakash, Jackie L. Whittaker, Marie Westby, Richard Sawatzky, Stirling Bryan, Kusum Soni, Kamaljit Sidhu, Santosh Powar, Laurie J. Goldsmith

**Affiliations:** 1 School of Population and Public Health, University of British Columbia, 2206 East Mall, Vancouver, British Columbia V6T1Z3 Canada; 2 Centre for Clinical Epidemiology & Evaluation, Vancouver Coastal Health Research Institute, 828 W 10Th Ave, British Columbia Vancouver, V5Z1M9 Canada; 3 Department of Physical Therapy, University of British Columbia, 212 Friedman Building, 2177 Wesbrook Mall, Vancouver, British Columbia V6T1Z3 Canada; 4 Arthritis Research Canada, 2238 Yukon St #230, Vancouver, British Columbia V5Y3P2 Canada; 5 Mary Pack Arthritis Program, Vancouver Coastal Health, 895 W 10Th Ave, Vancouver, British Columbia V5Z1L7 Canada; 6 Centre for Advancing Health Outcomes, Providence Health, 570-1081 Burrard Street, Vancouver, British Columbia V6Z1Y6 Canada; 7 School of Nursing, Trinity Western University, 22500 University Drive, Langley, British Columbia V2Y1Y1 Canada; 8 University of the Fraser Valley, 33844 King Road, Abbotsford, British Columbia V2S7M7 Canada; 9 Patient Research Partner, South Asian Exercise Research, Abbotsford, British Columbia Canada; 10 Faculty of Health Sciences, Simon Fraser University, 8888 University Drive, Burnaby, British Columbia V5A1S6 Canada

**Keywords:** Cross-cultural adaptation, KOOS, Knee injuries, Osteoarthritis, Punjabi

## Abstract

**Background:**

The Knee injury and Osteoarthritis Outcome Score (KOOS) is a knee-specific patient-reported outcome that is used to assess knee-related symptoms, function and quality of life across a variety of knee conditions in patient populations. Currently there is no Punjabi version of the tool available. This study aims to cross-culturally adapt the KOOS tool from the source English language to the target Punjabi language for use in the Canadian health context.

**Methods:**

We followed standard guidelines including: 1) creation of a concept definition document 2) forward translation 3) reconciliation 4) back translation 5) expert committee review 6) creation of pilot version for cognitive interviews 7) cognitive interviews 8) final review and proof reading.

**Results:**

Thirty people identifying as South Asian with lived experiences of various knee conditions took part in cognitive interviews (70% women, mean age 61 years) to provide insights into equivalence in conceptual, semantic, and content between the source English language and the target Punjabi language KOOS. Cognitive interviews identified comprehension and interpretation, structural, conceptual, cultural, and other issues in the preliminary Punjabi KOOS. These issues were addressed considering the Punjabi audience and culture in Canada, and the purpose of the tool to arrive at a cross-culturally adapted Punjabi KOOS.

**Conclusion:**

A cross-culturally Punjabi version of the KOOS is available to assess knee related outcomes of SA Punjabi patients in Canada. Future validation of the tool is required with SA Punjabi patients in Canada to ensure that the “target Punjabi instrument” has the same properties as the “original English KOOS instrument”.

**Supplementary Information:**

The online version contains supplementary material available at 10.1186/s12891-025-08870-y.

## Introduction

The Knee injury and Osteoarthritis Outcome Score (KOOS) is a patient-reported outcome instrument used to assess knee related outcomes in clinical research and practice. The KOOS was developed as an extension of the Western Ontario and McMaster Universities Osteoarthritis Index (WOMAC) [1] for people of all ages across a variety of knee conditions [1, 2]. In addition to the WOMAC, which assesses osteoarthritis (OA) specific domains (subscales) of Pain, Symptoms (e.g., stiffness), and Function in Activities of Daily Living (ADL), the KOOS assesses two additional domains (subscales): Function in Sports and Recreation (Sport/Rec), and Quality of Life (QOL) making it relevant to a range of knee injuries (e.g., ligament, meniscus injury or chondral injuries), conditions (e.g., OA) and orthopedic procedures (e.g., Anterior Cruciate Ligament (ACL) reconstruction and knee arthroplasty (TKA) [1–5]. A synthesis of 37 studies confirms acceptable psychometric properties of the KOOS across a range of knee conditions, and have found it to be more sensitive and responsive than WOMAC in younger or more active patients [6].

The KOOS was developed in US-English and Swedish languages (1994–95) and evaluated for test–retest reliability, construct validity, and responsiveness to clinical changes [1, 5, 7]. Since its development, the KOOS has been translated into more than 50 languages [5]. Currently, a Punjabi version of the KOOS is not available. Canada has over 2.3 million citizens reporting South Asian (SA) ethnicity [8], with 942,170 Punjabi-speaking people living in Canada [9]. Punjabi is one of the most spoken languages in Canada, ranking third after the two official languages of English and French [9]. Besides cultural differences in expression, some members of the SA population self-report poor health and low socioeconomic status, experience discrimination and culturally insensitive care and information, have limited English language proficiency, and are unfamiliar with local services compared to white populations [10]. A Punjabi version of the KOOS for Canada that is comparable to the English version would facilitate collection of data that is more accurate, interpretable, and generalizable, and make research more accessible to participants who might otherwise be excluded.

There are multiple approaches to instrument translation including literal translation, translation by a committee, and back translation [11]. To ensure that a translated instrument is culturally relevant and comprehensible while maintaining the meaning and intent of the original items, it is critical that the translation approach is appropriate and the validation rigorous. “Cross-cultural adaptation” is one approach that looks at both language (translation) and cultural adaptation issues in the process of preparing an instrument for use in another setting and population [12]. As cross-cultural adaptation is resource intensive, researchers sometimes use literal translations of English versions of previously validated instruments, without modifying the instrument in any major way, leading to “cultural hegemony” [11]. Our aim is to translate and cross-culturally adapt the KOOS tool to the Punjabi Canadian health context. We describe a cross-cultural adaptation process of the KOOS that is: 1) conceptually equivalent to the original English version; 2) culturally relevant to, and 3) easily understood by the Punjabi population with different knee conditions in Canada.

## Materials and method

**Fig. 1:**
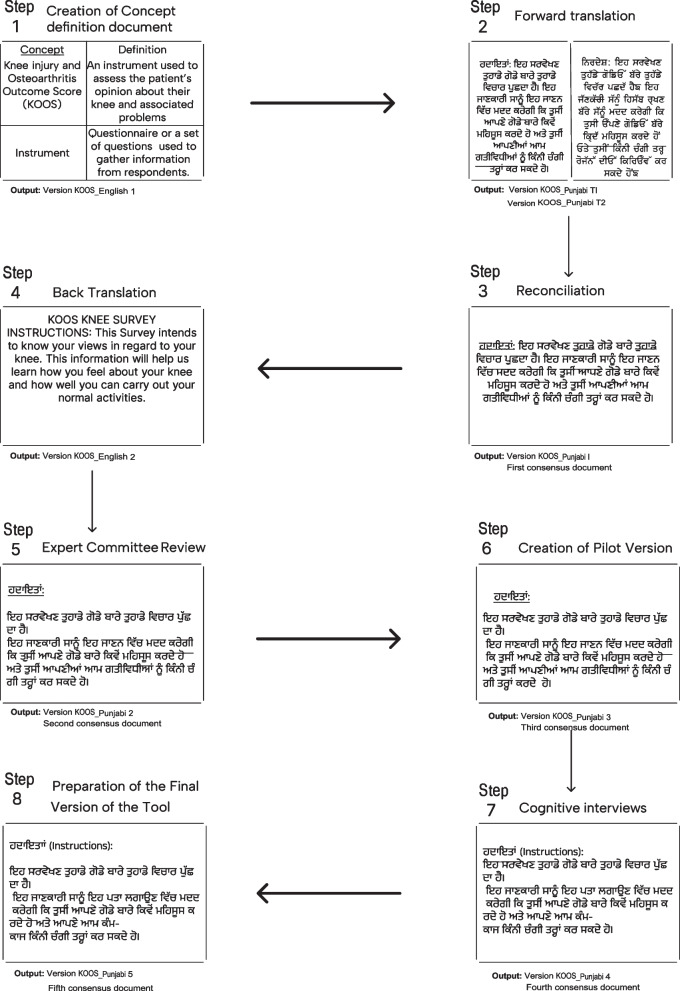
Pictorial flow chart of the translation of the original KOOS English instruments to Punjabi (Canadian)

After obtaining permission from the developer of the KOOS (Dr. Ewa Roos) to undertake the cross-cultural adaptation, we followed pre-established guidelines for instrument translation and adaptation recommended by the ISOQOL Translation and Cultural Adaptation Special Interest Group (TCASIG) [13]. This process included 8 steps (Fig. 1): 1) creation of a concept definition document, 2) forward translation, 3) reconciliation, 4) back translation, 5) expert committee review, 6) creation of pilot version, 7) cognitive interviews, and 8) final review and proofreading.

- Step 1: Creation of a concept definition document
  We created a concept definition document and shared it with the developer. This document contained item wise definitions of key words and phrases, and information about the conceptual basis for each item or task in the measure. The developer made no changes to this document.
- Step 2: Forward translation
  We hired two professional bilingual translators to forward translate the original KOOS-English (KOOS_English-1_) into Punjabi. Both translators had emigrated from India and were native Punjabi speakers. They had experience translating healthcare documents in Canada, but no clinical knowledge about knee conditions or OA. Translator #1 (TI) held a master’s degree in education and was working in healthcare research. Translator #2 (T2) held a diploma in Early Childhood Education and served as the project’s lay person to ensure that the Punjabi KOOS would be understood by the general population [12]. We provided translators with the concept definition document to ensure consistent understanding of KOOS concepts. Translators worked independently keeping track of the terms that they perceived as not appropriate in the SA context or difficult to translate such as ‘keeping track of’, ‘stockings’, ‘bed’, ‘bath’, ‘clicking of knee’, and ‘quality of life’ to be probed further during the cognitive interviews. This step produced two translations of the KOOS in Punjabi KOOS_Punjabi T1_ and KOOS_Punjabi T2_.
- Step 3: Reconciliation
  We created a synthesis of the two independent forward translations (KOOS_Punjabi T1_ and KOOS_Punjabi T2_). Translators discussed and reconciled discrepancies, and produced the first consensus version of the KOOS in Punjabi (KOOS_Punjabi-1_).
- Step 4: Back translation
  The KOOS_Punjabi-1_ was back translated to English (KOOS_English-2_) by a third, independent translator (T3) who was blinded to the original KOOS_English-1_ and concept definition document. Like T2, the third translator brought the lens of the general Punjabi Canadian community (i.e., no healthcare background).
- Step 5: Expert committee review
  The KOOS_English-2_ was reviewed by the developer and the study team (inclusive of physiotherapists, methodologists, a SA Punjabi speaking researcher, a SA non-Punjabi speaking researcher, and a Punjabi speaking patient research partner with lived TKA experience). The team critically analyzed the back translation to ensure appropriate terminology and semantic conceptual equivalence with the original KOOS_English-1_ was achieved. All team questions were resolved and the second consensus version of the KOOS in Punjabi (KOOS_Punjabi-2_) was produced.
- Step 6: Creation of pilot version
  Two Punjabi speaking team members prepared the tool for pretesting by proofreading KOOS_Punjabi-2_ and correcting grammatical errors. A third consensus version was developed (KOOS_Punjabi-3_).
- Step 7: Cognitive interviews
  We pre-tested KOOS_Punjabi-3_ using cognitive interviews. Cognitive interviewing is a psychologically oriented method for empirically studying how individuals mentally process and respond to survey questionnaires [14, 15]. It can also be defined as a means for applying qualitative research methods to the understanding of the functioning of questions [16]. Through this process we assessed the equivalence between the source (English) and target (Punjabi) language KOOS to determine if the variation of item interpretations between both was acceptable given the measurement goals [17].

### Cognitive interview participants and recruitment

We recruited a purposive sample of 30 participants (the recommended sample size for cross-cultural translation) [12, 18] to ensure a sample with diversity by gender, sex, age, education level, income, and health characteristics to promote the generalizability of the Punjabi KOOS among the Punjabi population in Canada. Participants were eligible to participate in the cognitive interviews if they were 19 years of age or older, self-identified as SA (i.e., ethnic origin from India, Pakistan, Sri Lanka, Bangladesh and related diaspora), were able to read and speak Punjabi, and had a traumatic knee injury that had caused damage to multiple knee structures (including ligament, meniscus or cartilage lesions), osteochondritis dissecans, knee OA, or undergone either an ACL reconstruction, knee arthroscopy, or TKA for OA more than 3-months previously. Participants were not eligible if they had rheumatoid arthritis or other inflammatory joint diseases, or if they had completed the KOOS in the past 3-months of this study.

Participants were recruited from the Greater Vancouver geographic region of British Columbia, Canada through existing networks and contacts at community groups and programs serving Punjabi people, through clinician’s offices, announcements on a SA radio station, and social media accounts of study team members.

### Cognitive interview procedures, data collection and analysis

Potential participant who expressed interest in the study were mailed/emailed a Punjabi study consent form. Participants were offered the opportunity to orally review the consent by phone. Interested participants provided written consent prior to the interview. Interviews were performed in Punjabi at participants’ homes, were audio recorded, and lasted approximately for 60 min. The interviews were conducted by two SA qualitative researchers knowledgeable of Punjabi culture, and fluent in Punjabi and English. Both interviewers received training before data collection by a qualitative methodologist on the team, including feedback on mock-data collection exercises. The interviewers worked together for the first two interviews to familiarize themselves with the probes and the cognitive interview process. They also performed self-reflection exercises after the interviews to discuss biases and prejudices that could influence the future interviews and data analysis process.

We used the Cognitive Interviewing Reporting Framework (CIRF) checklist to inform our cognitive interview methods [16]. We chose the *scripted verbal* and *emergent probing* technique for our cognitive interviews [14, 15, 19] given our experiences working with the SA population in Canada, and the advice provided by the patient research partners on the team. Verbal probing involves asking a series of probe questions to explore the thought processes of the participants [20]. Responding to probe questions (both scripted and emergent) are likely less challenging for our SA participants since SAs in general do not participate in research [21], and the cultural emphasis of humility in communication especially among the elderly population which might result in underreporting or biased responses. Moreover, there is evidence that scripted verbal and emergent probe techniques are effective cognitive interview methods among the SA population in Canada [17].

Verbal probing questions focused on issues of KOOS_Punjabi-3_ item comprehension, processing and match with the original KOOS_English-1_ concepts. During Steps 2–5 of the translation process we included probes for each KOOS item and additional probes for terms that were identified as not appropriate to the SA context ( “What do you think this [word] means? What word would you use to define [word] in Punjabi?”). A structured script for verbal probing was developed with a methodologist and the measurement expert team members to ensure we captured participant’s comprehension, retrieval, judgement and response to all items [22]. See Table 1 for a script of verbal probes used for KOOS items. We asked participants to choose alternate words or expressions if any word was not appropriate or confusing to ensure that the words used in the statements were culturally relevant. Verbal probes were asked immediately after a participant read and completed their response to a KOOS item. We also used emergent verbal probes to elicit information in instances when the participant hesitated, made some facial expression, or asked the interviewer a question. Emergent verbal probing is an effective method among a variety of cultural and language groups [17]. Interviewers recorded notes for each KOOS item by participant in a template developed for this purpose. Emergent verbal probes that were asked were also noted to be used during subsequent interviews if relevant and during the analysis.

**Table 1:** Scripted verbal probes used during cognitive interviews

- What do you think the question is asking? - What were you thinking when you answered the question? - Can you repeat this question in your own words? - Do you have any difficulty answering the question? - What do you think this [word to be probed] means? - Do you any suggestions to change the question? - What word would you use to define [words noted down during steps 2–5] in Punjabi? - Do you have any additional remarks?	

Three team members conducted the analysis of the cognitive interview data. One had knowledge about the research process and SA perspectives, and the other two had a firm grasp of English and Punjabi grammar and syntax, and were knowledgeable of the cultural and regional nuances of Punjabi. All three examined the data jointly, item by item across participants. This combination of expertise and joint collaboration was key to achieving a rigorous translation. A summary document for each KOOS item was developed and uploaded in NVivo 12 and common themes (issues and similarities) across all the items in the data explored. Interview audio recordings were used for clarification of notes about participant responses when needed. Items were revised if two or more interview participants identified an issue or interpreted the item in the way that did not fit with the original intent. Study team members reviewed the cognitive interviews findings and revised the translated tool accordingly and a fourth consensus version KOOS_Punjabi-4_ was developed. A final summary document of each KOOS item was sent to the developer for feedback and approval.

- Step 8: Preparation of the final version of the tool
  Two Punjabi speaking team members and the expert panel patient research partner completed the tool for understandability, typographical and layout errors. This resulted in the final Punjabi KOOS (KOOS_Punjabi-5_). Additional file 2 provides a sample page of the final Punjabi tool.

## Results

### Forward and backward translations (Steps 1–4)

When combining the two forward (KOOS_Punjabi T1_ and KOOS_Punjabi T2_) translations, we identified differences in technical words (e.g., “feel grinding”, “hear clicking of the knee”, “catching of the knee”) and common words and phrases (e.g., “keeping track of”) related to synonym use and translator Punjabi dialect differences. T1 provided “literal” word translations, while T2 was more “vernacular”. For example, T1 translated “clicking sounds” as “clicking” in Punjabi, while T2 wrote “tucktuck” in Punjabi. Similarly, T1 translated “first wakening in the morning”, as “wakening in the morning,” while T2 wrote “wakening early morning”. In both instances, the T2 version was accepted because it was a better fit in the given context. We reconciled all differences referring to the concept definition documents for the measurement goal of the item to produce KOOS_Punjabi-1_.

As expected, the backward translated KOOS_English-2_ differed from the original English KOOS due to the nature of language. Minor differences were synonym related (e.g., “difficulty” versus “trouble”, “rising from sitting” versus “getting up from sitting”). Conceptual differences included “lying” versus “sleeping”, “twisting” versus “rotating” and “during the last week” versus “during the past week”. Other differences were related to differences in grammar and vocabulary between the two languages; Punjabi has a subject-object-verb word order unlike the English language. For example, “Do you feel grinding, hear clicking or any other type of noise when your knee moves?” (Item S2) was forward and back translated as “When your knee moves, do you feel a grinding, clicking, or other kind of sound?”. All minor, conceptual, and grammar/vocabulary differences were discussed by the expert committee until consensus was reached.

### Cognitive interviews participant demographics

We interviewed 30 people (out of 35 people contacted) with various knee conditions (n = 3 ligament injury, n = 2 meniscal injury, n = 13 physician-diagnosed knee OA, n = 12 TKA) who self-identified as SA. All participants originated from the State of Punjab in India and had migrated to Canada over 10 years prior to the interview. Participants’ mean age was 61 years (minimum -maximum 24–90 years) and 21 (70%) identified as women. Participants’ education ranged from Grade 6 to Master’s degree/professional degree.

### Cognitive interview results

Themes that we identified during the analysis of the cognitive interviews were: comprehension and interpretation issues, structural issues, conceptual issues, cultural issues, and other issues. (Additional file 1 provides item wise details of the cognitive interview data as well as revisions made to items.)

#### Comprehension and interpretation issues

Many of the translated items were seen as clear and straightforward and participants responses to the probes matched the original concepts of interest (Items S1, S4, S5, P1,P4, P5, P6, A1, A2, A8, A10, SP1, SP2, and SP3). Other items were harder to comprehend and interpret because they incorporated technical terms that participants were unfamiliar with, even though a description of these terms were added to the KOOS_Punjabi-3_ for clarity. For example, participants didn’t know what the translated word for knee “catching” or “hanging up” (Symptom subscale S3) meant. There was also confusion about references to time (e.g., “last week”) and ‘level’ (e.g., “higher level”) in the instructions. For example, one participant questioned “*Are we talking about high energy activities? Or more time spent on activities.”*

Other items lacked context which added to the difficulty of understanding their intended meaning. For example, discussing “later in the day” (Stiffness subscale S7), one participant stated, *“Mid-morning my knee is not as stiff as it is by late evening, so I need some time context here to respond accurately.”*

Some of the items were confusing because they contained more than one implicit question. For example, Item A7 (getting in/out of car) under the Function, daily living subscale, was viewed as two items. A participant noted, *“Getting in a car is more difficult than getting out. How do I indicate that getting in was severe and getting out was moderate in this question?*” This person chose both “severe” and “moderate” as their answer. More examples are provided in the Additional file 1.

We addressed these issues of phrasing, ambiguity and readability using three approaches: (1) replacing a word with a more common word typically used by the Punjabi population in Canada; (2) describing a word keeping in mind the Punjabi dialects and idiomatic phrases used in Canada; or (3) inserting the original English word in brackets after the translated Punjabi word. For example, in the Punjabi KOOS all subscale titles (symptoms, stiffness, pain, functioning and quality of life) include both the translated Punjabi and original English words. One approach that we used sparingly was to define “troublesome” words in the Punjabi KOOS when there was no exact substitute Punjabi word (e.g., “kneeling” and “twisting”). The reason this approach was used sparingly was that it increases the length of the Punjabi translations which was not something desired by the interview participants.

#### Structural issues

A few of the translated items were very long and participants reported mental fatigue. We broke these items into shorter sentences for ease. Two participants also pointed out that it was difficult to differentiate between the categories “severely” and “extremely” (Quality of Life subscale Q2) and “severely” and “totally” (Quality of Life subscale Q3, Q4). Examples of structural issues and how they were resolved is summarized in the Additional file 1.

#### Conceptual issues

A few participants took “awareness of the knee problem” (Quality of Life subscale Q1) to mean *“Is there trouble in my knees?” or "Do you have knee pain*?”. Likewise, a few participants took “injured knee” (Functional, Sports and recreational activities subscale SP4) as the knee that has undergone surgery. These items were reviewed to see if the misinterpretations were due to the translation being too long, or if the item had an unsuitable connotation (i.e., unwanted associations of words), translation error, or had missing word(s) and we revised the item accordingly. Additional file 1 provides additional examples.

#### Culture specific issues

Some of the items were revised to make them more Canadian Punjabi culture specific. For example, with Function, daily living subscale items A9 and A11 (Putting on socks/stockings and taking off socks/stockings). The direct translation of “stocking” is not a common word among the Punjabi people as stockings are not fundamentally part of the SA attire. We deleted this word to avoid misunderstanding. There was some confusion whether the function, daily living subscale A13 (Getting in/out of bath*)* was about a “bathtub” or “shower” since both words are attributed to cleansing the body. We revised this statement to include bathtub and shower. Additional examples are provided in the Additional file 1.

#### Other issues

Issues were also noted with items that required to recall information, or that did not provide a “not applicable” option**.** Many times, participants forgot that they were to respond to the items thinking about their symptoms or pain in the last seven days. One technique recommended by them to address this and improve the accuracy of the responses, was by starting the item with the recall time. Similar recommendations were made for all the items under the pain subscale. However, the study team clinical and methodological experts decided that these changes would require revalidating the tool. Further, the overarching prompt at the start of the section would be redundant if this change was made.

Many items that involved vigorous movement were not scored by participants who had recently had a surgery. A “Not applicable” option was recommended by these participants. The KOOS developers have existing specific guidelines on how to address such issues when administering the tool. Additional file 1 provide some additional examples of other issues encountered in the cognitive interviews.

## Discussion

South Asian communities are the largest and fastest growing visible minority group in Canada [9], yet data on their health and well-being is limited due to various reasons including language barriers, limited understanding of the research process and the lack of translated and cross-culturally adapted research tools [23, 24].

We translated and cross-culturally adapted the original version of the KOOS tool into a Punjabi version using a multi-step process that included forward translation, back translation, expert review, and cognitive interviews, to enable assessment of knee related outcomes of SA Punjabi people in Canada. It has been argued that back translation may introduce errors, reinforce inaccuracies present in the original translation, reveal ambiguities in the translation that may lead to misinterpretations, and miss the naturalness and overall quality of the translated text [25–27]. Accordingly, studies adapting tools in SA languages often forgo back-translation [17] or use a multidisciplinary team-based discussion approach as an alternative to produce a conceptual translation of the questionnaire [28, 29]. However as back translation has been used in a majority of the KOOS cross cultural translations, including those in the last five years [30–35], we opted to use this standard translation method followed by cognitive interviews to ensure that the final translated survey is clear and understandable to the Punjabi speaking population in Canada.

SA cognitive interviews participants identified five types of issues with the KOOS_Punjabi-3_: comprehension and interpretation, structural, conceptual, cultural, and others. Participants reported that they had difficulties in understanding some of the technical translated words, some of the Punjabi language idioms and phrases, long translated items and concepts in some items especially under the KOOS “quality of life” subscale. Many of these challenges were due the literal translations especially of technical words during the forward and backward translation process which sometimes distorted the connotative meaning of the item in Punjabi due to the different grammatical and syntactical styles and terms between the two languages. We addressed all the difficulties pointed out during the cognitive interviews by replacing the technical terms with commonly used words recommended by the participants, or by describing the word or inserting the original English word in brackets after the translated Punjabi word. The final version of the tool was considered understandable and applicable by a SA patient partner on the team who represented future study participants and no further changes were deemed necessary.

These results are congruent with results from similar cross-cultural adaptation work of the KOOS, wherein modifications were necessary to the “original version” to improve the patient’s comprehension and understanding of items. For example, Item SP1 was modified from squatting to ‘crouch, squat’ to suit the Brazilian population [31]. Item A7 was revised from “Getting in/out of car” to “Riding in and getting off a vehicle” to suit the Filipino setting [36]; and Item S2 “Do you feel grinding, hear clicking or any other type of noise when your knee moves?” was revised as “Do you feel grinding/friction, hear clicking/cracking or any other noise when your knee moves?” as grinding” and “clicking” were not commonly used terms used by the Singapore population [37]. The Finnish cross-cultural adaptation work of the KOOS omitted the word “stockings” in Items A9 and A11 (“Putting on socks/stockings”, and “Taking off stocks /stockings”), and added the word “shower” as an alternative to bath in Item A13 (“Getting in/out of the bath”) [38]. These same items A9, A11, and A13 were replaced with South Asian culture specific items in the the Sinhalese translations of the KOOS [39]. There was difficulty in comprehension of Items P4 (“Bending knee fully) and translation of the category “ extreme” in Item Q4 in the Greek KOOS tool, resulting in discussions to select appropriate wording expressions [40]. The Arabic translation also transformed some items to capture the essence of the original concepts and make the questionnaire clear and understandable for the Egyptian population [41]. Although majority of the translations followed the Beaton et al. guideline [12] there is some heterogeneity in the number of forward translators from two [34] to three people [42]; the number of back translators from one [43] to three [41]; in the skill set of the translators from a mix of clinically trained and professional translators [44] to only professional language translators [45]; in the type of interviews from cognitive interviews [46] to interactive interviews [35], and in the composition of the expert team. In our study, we additionally created a concept definition document that was extremely useful in providing a clear roadmap for the forward translation process and reconciliation. We also actively engaged with a patient research partner throughout the process. In spite of these differences, all the studies have successfully obtained a comprehensible and culturally translated KOOS. Besides using an appropriate translation method that includes a translation verification and assessment step, careful and rigorous selection of translators who understand the cultural context, patient engagement on the team and ensuring sufficient time for the translation process are vital.

Based on our work, we emphasize the importance of having professional translators who are native speakers of the target language, fluent in the source language, and having experience in translation of instruments [27] with one of them preferably familiar with health-related terminology or the subject. Another learning is the importance of real-time documentation, capturing discrepancies and variations during the forward and back-translation process to further explore these issues during the cognitive interviews. Providing methodological training to culturally-sensitive interviewers to conduct the cognitive interviews resulted in high-quality capturing of the response of each item from participants in a quantitative and qualitative manner, while also ensuring that participants were at ease with the unfamiliar process of the interview. Critical review by experts on the team (physiotherapists, methodologists, experienced SA healthcare translators), as well feedback from the KOOS developer was very beneficial to ensure that the meaning of the translated items was the same as the original version, helping us produce a semantic and conceptual equivalent Punjabi translation of the KOOS tool.

We think it is important to allocate time to talk to the patient after they have filled in the translated tools to safeguard correct understanding of the items in spite of an “ideal” translation. Also explaining beforehand through an additional instruction sheet that some questions may seem irrelevant, or seem to contain more than one implicit question might also help avoid confusion. We also learnt in this study and through our experience working with the SA population in Canada, that SA seniors prefer to be interviewed rather than filling out a questionnaire. Researchers need to therefore consider adding more time and resources to collecting data using the KOOS and any other questionnaires from this population.

The next steps will involve clinical studies in patients with knee injury, knee OA and post-arthroplasty patients to evaluate the psychometric properties (e.g., validity, reliability and responsiveness) of the Canadian Punjabi KOOS with the objective of establishing psychometric comparability with the original instrument. Our focus on rigorous documentation at every step, constant communication and a consensus-building approach ensured improved translation and cross-cultural adaptation of the instrument. The availability of qualified translators, all with good understanding of the Punjabi culture in Canada, and an expert team of physiotherapists using the English KOOS instrument in their practice and SA researchers and methodologists contributed to the success of this work.

This version is limited to Punjabi patients in Canada and validation studies will be needed to determine performance with Punjabi people in India. Inadvertent interviewer bias could have influenced the qualitative data analysis, even though we engaged in critical reflection during the whole translation process. Other limitations include costs, especially the human resources and time required to conduct this work that hindered us from validating the tool.

While there is no denying the value of validation and analysis of psychometric properties, research papers focusing mainly on the psychometric validation due to word count limitations among other challenges, undermine the importance of the adapted translation [48, 49]. There is a need to push for more openness in describing the detailed translation methods to benefit future researchers and also enhance the quality of translated questionnaires [27]

## Supplementary Information

Additional file 1: Item wise revisions made to the KOOS items after the cognitive interviews. Revisions made to the original KOOS items based on the cognitive interview feedback.

Additional file 2: Sample pages of the final Punjabi tool. Sample copy of the KOOS tool translated into Punjabi. For the final copy and permission to use, contact Mapi Research Trust, Lyon, France, https://eprovide.mapi-trust.org.

## Data Availability

No datasets were generated or analysed during the current study.

## Abbreviations

KOOS: Knee injury and Osteoarthritis Outcome Score
WOMAC: Western Ontario and McMaster Universities Osteoarthritis Index
OA: Osteoarthritis
ADL: Activities of Daily Living
QOL: Quality of Life
ACL: Anterior Cruciate Ligament
TKA: Total Knee Arthroplasty
SA: South Asian
CIRF: Cognitive Interviewing Reporting Framework
