# Supplementary material for: Cross-cultural adaption of the Knee injury and Osteoarthritis Outcome Score (KOOS) into Punjabi for knee injury and osteoarthritis patients in Canada
Source: BMC Musculoskelet Disord. 2025 Jul 4;26:620. doi: 10.1186/s12891-025-08870-y (PMC12232004; doi:10.1186/s12891-025-08870-y)
Supplement: Supplementary file 1 — Additional file 1: Item wise revisions made to the KOOS items after the cognitive interviews. Revisions made to the original KOOS items based on the cognitive interview feedback. [file 12891_2025_8870_MOESM1_ESM.pdf]

Additional file 1: Revisions made to the KOOS items after the cognitive interviews

KOOS Source text	Cognitive Interview Data	Revision made to the item before and after the cognitive interview
**Original text: INSTRUCTIONS:** This survey asks for your view about your knee. This information will help us **keep track of** how you feel about your knee and how well you are able to perform your usual activities.<br><br>Punjabi text: **INSTRUCTIONS :** This survey asks your views about your knee. This information will help us **know** how you feel about your knee and how well you are able to perform your usual activities.	Punjabi words for instructions, and activities were not understood and alternate common words for these two words were proposed. Few participants were confused which knee they had to think about when responding to the questions because they had symptoms/problems with both knees. They wondered if it was possible to add a line indicating the knee – right or left.	We had replaced the word for “keep track of” with “know” during the forward translation phase prior to the cognitive interviews. We replaced the Punjabi word for instructions and activities with a Punjabi synonym proposed by many participants.
Original text: Answer every question by ticking the appropriate box, only <u>one</u> box for each question. If you are unsure about how to answer a question, please give the best answer you can.<br><br>Punjabi text: Answer every question by ticking only <u>one</u> appropriate square. If you are not fully sure about your answer, please answer what you think is right.	According to some Punjabi participants, the word “box” could also mean packages, containers, suitcases or cartons. They suggested this word with ਖਾਨੇ "Khane" (“squares”) as this is what is used in the voting ballets in Canada. A few also felt that the last sentence was not clear.	We simplified the instruction to make it shorter and easier for the patients to understand and replaced “box” with “square”.
Original text: **Symptoms**<br><br>Punjabi text: ਲੱਛਣ (Symptoms)	Some participants didn’t understand the Punjabi word “Lachaṇa” for symptoms. There was no alternate word proposed that was appropriate.	We decided to keep “Lachana” and add “Symptoms” within brackets instead of defining this word to avoid lengthy texts.
Original text: These questions should be answered thinking of your knee symptoms during the **last week.**<br><br>Punjabi text: These questions below should be answered keeping in mind your knee symptoms during <u>**the last seven days**</u>	A few participants were confused about the time period since the week starts on Monday and ends on a Sunday. One participant said, “ *Today is Saturday, so it is not a full week. Should I think about these questions from Monday or the full week before that*?”<br><br>Many times, participants forgot that they had to respond to the questions thinking about their symptoms last week. It was suggested that we remind them in every sentence. One participant suggested, “*If you start the question with last week, we might remember. Otherwise we will respond to the question in a general way. "*	The expert team decided to change “last week” to the “last seven days” so that there is no confusion in the minds of participants filling out the tool and it refers to the same time period in everyone’s mind- the last seven days preceding the current date.<br><br>We did not make any other changes that would require revalidating the tool.

Original text: S2. Do you feel grinding, hear clicking or any other type of noise when your knee moves?<br><br>Punjabi text: S2: When your knee moves, do you feel grinding, or hear clicking or any other noise?	A few of our interview participants didn't know what the Punjabi word for grinding "Pīsaṇā." meant. We had to explain the word to them. A few of our participants also did not understand what knee clicking meant.	We went with a more common word for clicking " tucktuck" during the forward translation. We replaced "Pīsaṇā" with "Ragarana" which is a synonym for the word that was recommended during the interviews.
Original text: S3. Does your knee catch or hang up when moving?<br><br>Punjabi text: S3: When you move, does your knee get stuck or jammed?	Some study participants did not know what the Punjabi words for "knee catching" and "hanging up" meant. After the interviewer explained it to them, they suggested using Punjabi words that translated to "getting stuck" "locked" or "jammed".	We replaced Latakdha with ਜਾਂ ਜਾਮ ""“jāṁ jāma" in Punjabi.
Original text: **Stiffness**<br><br>Punjabi text: **ਅਕੜੇਵਾਂ (Stiffness)**	A few participants did not understand the Punjabi word for stiffness.	We included the word stiffness within brackets after "Akaddhevaan" similar to what we did in the previous section (symptoms)
Original text: The following questions concern the amount of joint stiffness you have experienced during the **last week** in your knee. Stiffness is a sensation of restriction or slowness in the ease with which you move your knee joint.<br><br>Punjabi text: The questions below concern the amount of stiffness you have experienced in your knee during **the last seven days**. Stiffness is something that makes it hard to move or slows down the movement of your knee joint.	Most participants didn't understand the Punjabi translation of the stiffness definition. They did not understand the Punjabi words for "sensation, or "restriction". They wanted an easier definition or suggested removing the definition.	We revised and explained the stiffness definition in layman's terms based on the feedback participants provided during the interview.
Original text: S6. How severe is your knee joint stiffness after first wakening in the morning?<br><br>Punjabi text: S6: When you wake up in the morning, how stiff is your knee?	A few participants suggested deleting the word severe or " gambeer" from the sentence because the options for this question are severe , extreme, moderate etc.	We have deleted the word "severe" from the item.
Original text: S7. How severe is your knee stiffness after sitting, lying or resting later in the day?<br><br>Punjabi text: S7: How stiff is your knee joint after sitting, lying or resting during the day?	There was discussion during the interviews about what later in the day could mean with many participants understanding it as mid-morning until late evening	We revised the sentence to the end of the day first and after discussion with the developer to during the day which covers the duration after awakening in the morning.

Original text: What amount of knee pain have you experienced the last week during the following activities?<br><br>Punjabi text: What amount of knee pain have you experienced during **<u>the last seven days</u>** while doing the following activities?	The Punjabi words for “activities” and “experience” were considered bookish and not easily understandable especially by our senior participants.	We replaced the Punjabi words for “activities” and “experience” with common everyday language.
Original text: P2. Twisting/pivoting on your knee<br><br>Punjabi text: P2: Twisting your knee while standing, without moving your foot	Participants found it difficult to explain this question in their own words. There is no single word for “pivoting” in Punjabi with the word used for “twisting” also used for “pivoting”. Twisting and pivoting were defined as moving from one side to another, moving your knee without moving your foot. One participant also suggested adding “knee pain” to all these questions for further clarity.	We used one word in Punjabi to depict both twisting and pivoting and described the twisting action.
Original text: P5. Walking on flat surface<br>Punjabi text: P5. While walking on flat surface	A few participants proposed replacing the current word used in the tool for flat surface with a more commonly used term.	We replaced the Punjabi word for flat surface with a synonym that was recommended during the interviews.
Original text: **Function, daily living**<br><br>Punjabi text: **ਕਾਰਜ, ਰੋਜ਼ਾਨਾ ਜੀਵਨ (Function, daily living)**	The exact translation of.ਕਾਰਜ, ਰੋਜ਼ਾਨਾ ਜੀਵਨ is Activities of daily life.	We included the original English word within brackets as we did with the previous subscales
Original text: The following questions concerns your physical function. By this we mean your ability to move around and to look after yourself.<br><br>Punjabi text: The questions below concern the degree of difficulty you have experienced due to your knee carrying out the following physical activities during **<u>the last seven days.</u>** By physical activities we mean your ability to move around and look after yourself.	Many found the instructions hard to repeat in their own words and the translated sentence too long. Some participants wanted the Punjabi word for function replaced with a simpler word.	We have simplified the sentence and we highlighted last seven days in this section to keep it uniform with the other sections.
Original text: For each of the following activities please indicate the degree of difficulty you have experienced in the **last week** due to your knee.<br><br>Punjabi text: Please describe during **<u>the last seven days,</u>** how much	Similar to what we heard under the pain subscale instructions, the Punjabi words for “activities” and “experience” were considered bookish and not easily understandable .	We replaced the Punjabi words for “activities” and “experience” with common everyday language.

difficulty did you experience due to your knee while performing the following activities.		
Original text: A8. Going shopping<br>Punjabi text: A8: While going shopping	A few participants wanted shopping to be put within brackets for clarity. Otherwise it was an easy item to understand, repeat and complete.	We inserted " shopping" after the Punjabi word "ਖਰੀਦਦਾਰੀ" (Kareedhdhari) in brackets.
Original text: A9 and A11. Putting on socks/stockings/Taking off socks/stocking<br><br>Punjabi text: A9/A11: While putting on socks/ While taking off socks	The word "stockings" is not used by many SA. Some SA called it "long socks". Some participants recommended deleting this word to avoid any potential misunderstanding while reading this statement.	We deleted the word stocking from the item.
Original text: A12. Lying in bed (turning over, maintaining knee position)<br><br>Punjabi text: A12: While lying in bed, turning over without moving your knee	Some participants reported that the question was complicated. Many were unable to describe "maintaining knee position in bed" in their own words. One participant queried, "*Is this question asking about pain while turning over in bed, is it asking can I turn over in bed? What is it asking?"*	We revised " maintaining knee position" to "turning over without moving your knee".
Original text: A13. Getting in/out of bath<br>Punjabi text: A13. While getting in/out of the bathtub/shower	Bath for many SA participants included both washing the body in a tub or shower.	We included both bathtub and shower in the item.
Original text: A14. Sitting<br>Punjabi text:<br><br>A14: While sitting (chair, floor etc.)	A few participants questioned whether it is sitting on a chair or on the floor.<br>Quote: "*Are we talking about sitting on a chair or the floor? I guess it is a chair. Because we sit down on the floor also. Also , write "lamba" time (long time) because there is difficulty if I sit for too long versus just sit for a few minutes*. "	We included chair, floor etc within brackets to make clear it could be sitting anywhere.
Original text: For each of the following activities please indicate the degree of difficulty you have experienced in the **last week** due to your knee.<br><br>Punjabi text: Please describe, during **<u>the last seven days</u>**, while performing the following activities, how much difficulty did you experience due to your knee.	A few participants recommended changing the tense in Punjabi from "Hoiyan" to "Hoi" and proposed some simplified versions of the Punjabi sentence.	We have simplified the instructions and made it short and easier to read.

Original text: A16. Heavy domestic duties (moving heavy boxes, scrubbing floors, etc)<br><br>Punjabi text: A16: While doing heavy domestic duties (for example carrying heavy shopping bags, carrying small children etc.)	Many participants go shopping, take care of their grandkids and said that lifting them, giving them a bath is heavy work.	We have included some SA specific examples within brackets
Original text: A17. Light domestic duties (cooking, dusting, etc)<br><br>Punjabi text: A17: While doing light domestic duties (for example cooking, house cleaning etc.)	Dusting as a word was not easily relatable to a few participants though they said that they clean the house and dust in the process.	We changed dusting to "Ghad Pouncha" or "house-cleaning".
Original text: The following questions concern your physical function when being active on a higher level. The questions should be answered thinking of what degree of difficulty you have experienced during the **last week** due to your knee.<br><br>Punjabi text: The questions below are about your vigorous physical activities. Answer the questions thinking about the degree of difficulty you have experienced due to your knee during the **last seven days.**	Participants wanted a simpler, shorter instruction from the long-translated instruction.	We simplified the instructions
Original text: SP4. Twisting/pivoting on your injured knee<br><br>Punjabi text: SP4. While twisting on your affected knee without moving your foot	One participant asked, *"How do you define an injured knee?"* A few participants recommended changing the Punjabi word "Jhakme" (injured) to "Dukdhe" (painful) knee. Injured to these participants meant they were hurt and had a wound that had to be surgically operated.<br>A not-applicable category was also recommended for this item.	We used one word to depict both twisting and pivoting and described the twisting action as done earlier. Injured knee was described as affected knee.
Original text: SP5. Kneeling<br><br>Punjabi text: SP5: While sitting on your knees	We had translated kneeling as ਗੋਡੇ ਟੇਕਦਿਆਂ (bowing down on the knee) prior to the cognitive interviews. Many Punjabi participants however preferred and recommended that we explain the action as sitting on your knees as they do in the temple or the forehead touching the ground.<br><br>Some participants also pointed out the need to include a "Not applicable" category to this question.	We went with sitting on your knees as it flowed better in Punjabi to describe kneeling.

Original text: Quality of Life<br>Punjabi text: **ਜੀਵਨ ਸ਼ੈਲੀ (**Quality of Life)	No participant liked the Punjabi word "Gunnavatta" which is the exact translation for quality of life. Some participants recommended using quality of life as they didn't know how to translate this in Punjabi.	We have used the word Jeevan Shelley that was used by many participants which translates to Lifestyle quality.
Original text: Q1. How often are you aware of your knee problem?<br><br>Punjabi text: Q1. How often do you think about or feel your knee problem?	Most participants wanted the question to be rephrased in a way that it made sense to them. Some could not repeat the sentence in their own words. Some interpreted the sentence as , "*How many times do you think about your knee?" or "Is there trouble in my knees*?". One person recommended changing the options to Zyada/ thoda (a lot/little) as they are thinking of knee problems as knee pain.	We replaced 'aware' with 'feel' and 'think'.
Original text: Q2. Have you modified your life style to avoid potentially damaging activities to your knee?<br><br>Punjabi text: Q2. Have you modified your lifestyle to prevent your knee from potential damaging activities?	Many participants said the question did not make sense and was confusing but they were able to interpret the question. We examined the different interpretations of this sentence and used it for our revision.	We simplified the sentence
Original text: Q3. How much are you troubled with lack of confidence in your knee?<br>Punjabi text:<br><br>Q3. How much trust do you have in your knee?	"Troubled with lack of confidence" was the most difficult part of the sentence to understand for many participants. We had a number of different interpretations	We discussed all the recommendations that were provided and went with "trust" that we felt was the closest to the construct of " lack of the confidence" and the easiest to understand in Punjabi.
