# Supplementary material for: Cross-cultural adaption of the Knee injury and Osteoarthritis Outcome Score (KOOS) into Punjabi for knee injury and osteoarthritis patients in Canada
Source: BMC Musculoskelet Disord. 2025 Jul 4;26:620. doi: 10.1186/s12891-025-08870-y (PMC12232004; doi:10.1186/s12891-025-08870-y)
Supplement: Supplementary file 2 — Additional file 2: Sample pages of the final Punjabi tool. Sample copy of the KOOS tool translated into Punjabi. For the final copy and permission to use, contact Mapi Research Trust, Lyon, France, https://eprovide.mapi-trust.org. [file 12891_2025_8870_MOESM2_ESM.pdf]

‘ਕੂਸ’ ਗੋਡਿਆਂ ਦਾ ਸਰਵੇਖਣ (KOOS KNEE SURVEY)

ਅੱਜ ਦੀ ਮਿਤੀ: ____/_____/_____ ਜਨਮ ਮਿਤੀ: ____/_____/_____

ਨਾਮ: ____

ਹਦਾਇਤਾਂ (Instructions):

ਇਹ ਸਰਵੇਖਣ ਤੁਹਾਡੇ ਗੋਡੇ ਬਾਰੇ ਤੁਹਾਡੇ ਵਿਚਾਰ ਪੁੱਛਦਾ ਹੈ। ਇਹ ਜਾਣਕਾਰੀ ਸਾਨੂੰ ਇਹ ਪਤਾ ਲਗਾਉਣ ਵਿੱਚ ਮਦਦ ਕਰੇਗੀ ਕਿ ਤੁਸੀਂ ਆਪਣੇ ਗੋਡੇ ਬਾਰੇ ਕਿਵੇਂ ਮਹਿਸਸ ਕਰਦੇ ਹੋ ਅਤੇ ਆਪਣੇ ਆਮ ਕੰਮ-ਕਾਜ ਕਿੰਨੀ ਚੰਗੀ ਤਰ੍ਹਾਂ ਕਰ ਸਕਦੇ ਹੋ।

ਹਰੇਕ ਸਵਾਲ ਦਾ ਜਵਾਬ ਦੇਣ ਲਈ **ਸਿਰਫ਼ ਇੱਕ** ਢੁੱਕਵੇਂ ਖਾਨੇ ਤੇ ਨਿਸ਼ਾਨ ਲਗਾਉ। ਜੇ ਤੁਹਾਨੂੰ ਆਪਣੇ ਜਵਾਬ ਬਾਰੇ ਪੂਰਾ ਯਕੀਨ ਨਹੀਂ ਹੈ ਤਾਂ ਕਿਰਪਾ ਕਰਕੇ ਉਹ ਜਵਾਬ ਦਿਓ ਜੋ ਤੁਹਾਨੂੰ ਸਭ ਤੋਂ ਠੀਕ ਲੱਗਦਾ ਹੈ।

ਲੱਛਣ (Symptoms)

ਹੇਠਾਂ ਲਿਖੇ ਸਵਾਲਾਂ ਦੇ ਜਵਾਬ **ਪਿਛਲੇ ਸੱਤ ਦਿਨਾਂ ਦੌਰਾਨ** ਤੁਹਾਡੇ ਗੋਡੇ ਦੇ ਲੱਛਣਾਂ ਨੂੰ ਧਿਆਨ ਵਿੱਚ ਰੱਖਦੇ ਹੋਏ ਦਿੱਤੇ ਜਾਣੇ ਚਾਹੀਦੇ ਹਨ।

S1. ਕੀ ਤੁਹਾਡੇ ਗੋਡੇ ਵਿੱਚ ਸੋਜ ਹੈ?

ਕਦੇ ਨਹੀਂ	ਘੱਟ ਹੀ	ਕਦੇ ਕਦੇ	ਅਕਸਰ	ਹਮੇਸ਼ਾ
☐	☐	☐	☐	☐

S2. ਜਦੋਂ ਤੁਹਾਡਾ ਗੋਡਾ ਹਿੱਲਦਾ ਹੈ ਤਾਂ ਕੀ ਤੁਹਾਨੂੰ ਰਗੜਨ, ਟੱਕ-ਟੱਕ ਕਰਨ ਜਾਂ ਕਿਸੇ ਹੋਰ ਕਿਸਮ ਦੀ ਅਵਾਜ਼ ਮਹਿਸਸ ਹੁੰਦੀ ਹੈ?

ਕਦੇ ਨਹੀਂ	ਘੱਟ ਹੀ	ਕਦੇ ਕਦੇ	ਅਕਸਰ	ਹਮੇਸ਼ਾ
☐	☐	☐	☐	☐

S3. ਜਦੋਂ ਤੁਸੀਂ ਹਿੱਲਦੇ ਹੋ ਤਾਂ ਕੀ ਤੁਹਾਡਾ ਗੋਡਾ ਅੜਦਾ ਜਾਂ ਜਾਮ ਹੁੰਦਾ ਹੈ?

ਕਦੇ ਨਹੀਂ	ਘੱਟ ਹੀ	ਕਦੇ ਕਦੇ	ਅਕਸਰ	ਹਮੇਸ਼ਾ
☐	☐	☐	☐	☐

S4. ਕੀ ਤੁਸੀਂ ਆਪਣਾ ਗੋਡਾ ਪੂਰੀ ਤਰ੍ਹਾਂ ਸਿੱਧਾ ਕਰ ਸਕਦੇ ਹੋ?

ਹਮੇਸ਼ਾ	ਅਕਸਰ	ਕਦੇ ਕਦੇ	ਘੱਟ ਹੀ	ਕਦੇ ਨਹੀਂ
☐	☐	☐	☐	☐

Sample copy, do not use without permission

SP5. ਗੋਡਿਆਂ ਭਾਰ ਬੈਠਣ ਵੇਲੇ

ਬਿਲਕੁਲ ਨਹੀਂ	ਹਲਕਾ	ਮੱਧਮ	ਬਹੁਤ ਜ਼ਿਆਦਾ	ਅਤਿ ਦਾ
☐	☐	☐	☐	☐

ਜੀਵਨ ਸ਼ੈਲੀ (Quality of Life)

Q1. ਤੁਸੀਂ ਆਪਣੇ ਗੋਡੇ ਦੀ ਸਮੱਸਿਆ ਬਾਰੇ ਕਿੰਨੀ ਵਾਰ ਸੋਚਦੇ ਜਾਂ ਮਹਿਸੂਸ ਕਰਦੇ ਹੋ?

ਕਦੇ ਨਹੀਂ	ਮਹੀਨੇ ਵਿੱਚ ਇੱਕ ਵਾਰ	ਹਫਤੇ ਵਿੱਚ ਇੱਕ ਵਾਰ	ਰੋਜ਼ਾਨਾ	ਲਗਾਤਾਰ
☐	☐	☐	☐	☐

Q2. ਕੀ ਤੁਸੀਂ ਆਪਣੇ ਗੋਡੇ ਨੂੰ ਨੁਕਸਾਨ ਪਹੁੰਚਾਉਣ ਵਾਲੇ ਕੰਮ-ਕਾਜਾਂ ਤੋਂ ਬਚਾਉਣ ਲਈ ਆਪਣੇ ਜ਼ਿੰਦਗੀ ਦੇ ਤਰੀਕਿਆਂ ਵਿੱਚ ਕੋਈ ਤਬਦੀਲੀ ਲਿਆਂਦੀ ਹੈ?

ਬਿਲਕੁਲ ਵੀ ਨਹੀਂ	ਹਲਕਾ	ਮੱਧਮ	ਬਹੁਤ ਜ਼ਿਆਦਾ	ਪੂਰੀ ਤਰ੍ਹਾਂ
☐	☐	☐	☐	☐

Q3. ਤੁਸੀਂ ਆਪਣੇ ਗੋਡੇ ਉੱਤੇ ਭਰੋਸਾ ਨਾ ਹੋਣ ਕਾਰਨ ਕਿੰਨੇ ਪਰੇਸ਼ਾਨ ਹੋ?

ਬਿਲਕੁਲ ਨਹੀਂ	ਹਲਕਾ	ਮੱਧਮ	ਬਹੁਤ ਜ਼ਿਆਦਾ	ਅਤਿ ਦਾ
☐	☐	☐	☐	☐

Q4. ਆਮ ਤੌਰ 'ਤੇ ਤੁਹਾਡੇ ਗੋਡੇ ਕਾਰਨ ਤੁਹਾਨੂੰ ਕਿੰਨੀ ਕੁ ਮੁਸ਼ਕਲ ਆਉਂਦੀ ਹੈ?

ਬਿਲਕੁਲ ਨਹੀਂ	ਹਲਕਾ	ਮੱਧਮ	ਬਹੁਤ ਜ਼ਿਆਦਾ	ਅਤਿ ਦਾ
☐	☐	☐	☐	☐

ਇਸ ਪ੍ਰਸ਼ਨਾਵਲੀ ਵਿੱਚ ਸਾਰੇ ਪ੍ਰਸ਼ਨਾਂ ਨੂੰ ਪੂਰਾ ਕਰਨ ਵਾਸਤੇ ਤੁਹਾਡਾ ਬਹੁਤ ਬਹੁਤ ਧੰਨਵਾਦ।

Sample copy, do not use without permission

KOOS contact information and permission to use: Mapi Research Trust, Lyon, France, https://eprovide.mapi-trust.org
